# Sample Entropy, Univariate, and Multivariate Multi-Scale Entropy in Comparison with Classical Postural Sway Parameters in Young Healthy Adults

**DOI:** 10.3389/fnhum.2017.00206

**Published:** 2017-04-26

**Authors:** Clint Hansen, Qin Wei, Jiann-Shing Shieh, Paul Fourcade, Brice Isableu, Lina Majed

**Affiliations:** ^1^Research Department, Aspetar Qatar Orthopaedic and Sports MedicineDoha, Qatar; ^2^School of Information Engineering, Wuhan University of TechnologyWuhan, China; ^3^Department of Mechanical Engineering, Yuan Ze UniversityChung-Li, Taiwan; ^4^URCIAMS – Motor Control and Perception Team, University Paris-SudOrsay, France; ^5^PSYCLE, Aix Marseille UniversityAix-en-Provence, France; ^6^Sport Science Program, College of Arts and Science, Qatar UniversityDoha, Qatar

**Keywords:** center of pressure, sample entropy, multi-scale entropy, multivariate multi-scale entropy, visuo-kinesthetic effect

## Abstract

The present study aimed to compare various entropy measures to assess the dynamics and complexity of center of pressure (COP) displacements. Perturbing balance tests are often used in healthy subjects to imitate either pathological conditions or to test the sensitivity of postural analysis techniques. Eleven healthy adult subjects were asked to stand in normal stance in three experimental conditions while the visuo-kinesthetic input was altered. COP displacement was recorded using a force plate. Three entropy measures [Sample Entropy (SE), Multi-Scale Entropy (MSE), and Multivariate Multi Scale Entropy (MMSE)] describing COP regularity at different scales were compared to traditional measures of COP variability. The analyses of the COP trajectories revealed that suppression of vision produced minor changes in COP displacement and in the COP characteristics. The comparison with the reference analysis showed that the entropy measures analysis techniques are more sensitive in the incremented time series compared to the classical parameters and entropy measures of original time series. Non-linear methods appear to be an additional valuable tool for analysis of the dynamics of posture especially when applied on incremental time series.

## Introduction

Postural control is of paramount importance to ensure safe completion of complex tasks such as locomotion or simpler tasks such as standing. Postural control in healthy subjects is strongly affected by spatial orientation (Isableu et al., 1998) which in turn is based on both vestibulo-proprioceptive and visual cues (Asch and Witkin, 1948). Some subjects show an increased visual dependency (Guerraz et al., 2001) and therefore closing the eyes or introducing distorted visual feedback (e.g., wearing translucent goggles) increases the difficulty of standing tasks (Bronstein, 1986). A diminished postural control may result in a loss of equilibrium and consequently in falling (Carroll and Freedman, 1993; Collins and De Luca, 1993). This diminution may either be due to normal ageing (Pascolo et al., 2005, 2006) or due to a neurodegenerative disorder such as Parkinson's disease (Sabatini, 2000; Duarte and Zatsiorsky, 2001).

Usually, the quantification of postural control is included in clinical assessments but this strongly depends on the technology available. Either the postural control is rated based on clinical rating scales (Bloem et al., 2016) e.g., Tinetti Balance Scale, Rating Scale for Gait Evaluation, or on specific equipment such as force plates (Yamada, 1995; Newell et al., 1997) to measure body sway. The displacement of the center of pressure (COP) (the point of application of the vertical resultant force acting on the body from the supporting surface, dos Santos, 2008) is commonly measured when evaluating the postural control of a person (Carroll and Freedman, 1993; Collins and De Luca, 1993). The COP is highly irregular and non-stationary which has led multiple studies to characterize the functional effects of conditions such as disease, aging, cognitive task and visual perception on the postural stability (Ramdani et al., 2009).

Techniques for quantifying the displacement of the COP vary from descriptive measures (mean or standard deviation) to techniques taken from signal processing (root-mean-square and frequency analyses). The non-linear deterministic methods allow for the exploration of the randomness or predictability of the COP fluctuations. Those methods were used in studies investigating the deterministic features of COP dynamics (Myklebust et al., 1995; Riley et al., 1999; Doyle, 2004; Doyle et al., 2005), and their potentially chaotic behavior (Cavanaugh, 2005; Costa et al., 2005; Roerdink et al., 2006; Cavanaugh et al., 2007) but also on the quantification of the complexity of COP time series (Ramdani et al., 2009). Entropy family, as a non-linear measure of time series, has been widely applied to study features of COP displacement in different situations.

Entropy family quantifies the regularity (predictability) of a signal, with predictable (e.g., periodic) signals resulting in low entropy, or completely unpredictable signals, resulting in high entropy. A more regular COP pattern indicates that the postural behavior is more rigid (Donker et al., 2007), suggesting that the regularity of COP displacements and the amount of attention paid to postural control are dependent (Donker et al., 2007; Vuillerme and Nafati, 2007). Complexity on the other hand is associated with meaningful structural richness (Grassberger, 1991) incorporating correlations over multiple spatio-temporal scales. A decrease of complexity is related with a functional decline; and a more rigid postural behavior results in dysfunctional balance control during perturbations (Schniepp et al., 2013).

For instance, the Sample Entropy (SE) (Roerdink et al., 2006) has been used to investigate the effect of visual perception (Sabatini, 2000), cerebral concussion (Cavanaugh, 2005; Cavanaugh et al., 2007) and cognitive tasks (Wei et al., 2012) on postural dynamics. Multi-scale Entropy (MSE) and Multivariate Multi-Scale Entropy (MMSE) (Ahmed and Mandic, 2011) are both able to represent the complexity of non-linear time series in different scales (Ahmed and Mandic, 2011). They measure and quantify the intrinsic complexity (Costa et al., 2005) of single and multi-channel signals and then provide a meaningful measure of regularity in biological signals as e.g., COP measurements (Ahmed and Mandic, 2012).

To extract additional features of the COP displacement, the analysis of incremented time series of original signals may be useful (Ramdani et al., 2009) and this approach provides extra information compared to other approaches (Huang et al., 2013). Incremented time series, equivalent to the velocity of the COP displacements have been shown to be effective in the analysis of physiological signals (Costa et al., 2007). In this study two types of incremented time series were computed and studied, the relationship between anterior-posterior (A/P) and medio-lateral (M/L) COP displacement while using univariate and bivariate entropy measures. Although previous studies (Costa et al., 2007; Ramdani et al., 2009) applied incremental time series in their COP analysis, no proof has been provided to determine its performance and advantage.

The working hypothesis was to test if non-linear entropy family methods quantify COP displacement better when using incremented time series during three standing conditions with varying visuo-kinesthetic input. In addition to the entropy measures, classical posturographic methods were also used to assess the COP displacement. The classical methods were then compared with the entropy measures of the original time series.

## Methods experiments and subjects

Eleven healthy subjects (Mean age: 25.6 years; Mean bodyweight 73.76 kg) voluntarily participated in the experiment after signing a statement of informed consent as required by the Helsinki declaration and the Paris-Saclay STAPS (Sciences and Techniques of Sports and Physical Activities) local Ethics Committee. Subjects stood upright looking forward, on a force plate (AMTI OR6001200, Watertown, MA, USA) with their arms hanging loosely by their sides and feet separated by a self-selected distance (typically approximately 10 cm) for 30 s. Foot position was marked with an erasable marker to ensure the position was maintained if subjects needed to step off the force plate between trials or conditions. Subjects performed three trials of stationary bipedal standing in three different sensory conditions, eyes open (EO), eyes closed (EC) and perturbed vision (PV) by wearing translucent goggles, resulting in a total of nine trials. The trials for each condition were averaged accordingly.

**Figure 1:**
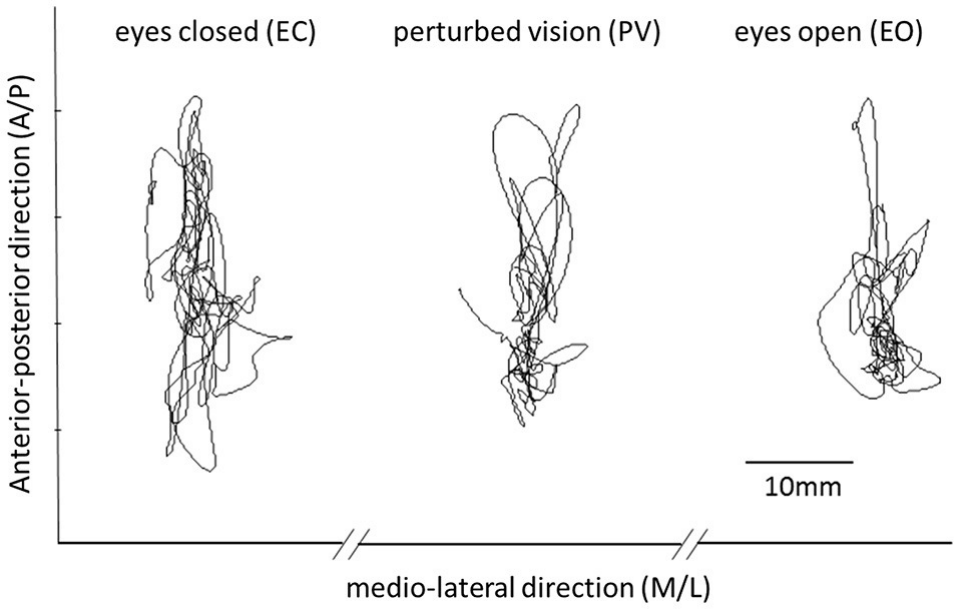
**This figure shows representative time series of COP data in each of the three experimental conditions (EO, EC, PV)**. Differences between conditions are explored using both classical parameters and entropy based methods.

In the PV condition, even though the eyes were opened, the visual input did not constitute a valid and meaningful source (i.e., neither self-motion nor self-orientation cues) of spatial referencing for postural balance, and cannot be integrated together with proprioception in an optimal manner (Jeka et al., 2000; Oie et al., 2001). Conditions were presented in a random order for each participant. Subjects were instructed to stand as still as possible with either the eyes open, eyes closed or wearing translucent goggles. The COP trajectories were recorded in both A/P and M/L directions at a sampling frequency of 250 Hz as shown in Figure 1. Rest periods of 60 s were provided between trials. The resulting data was low-pass filtered at 5 Hz using an 8th order Butterworth filter with a zero-phase digital filter (filtfilt.m) in the Matlab (MathWorks, Inc., USA) environment.

## Analysis

### Classical methods

To quantify differences between the conditions (EC, PV, EO), the following variables were calculated from the postural sway: the root-mean square (RMS), mean velocity (MV), path length (PL), mean frequency (MF) and the surface area (SA) of COP displacements in A/P and M/L directions. The RMS was defined as the quadratic mean and is a measure of the postural sway displacement in both the A/P and M/L directions [mm]. The mean velocity defines the mean velocity of the postural sway representing the path length divided by the trial duration [mm/s]. The path length was defined as the length of the COP trajectory displacements on the platform surface [mm]. The MF is the mean frequency of the power spectrum of the postural sway in both the A/P and M/L directions. The SA is the surface area given by the 95% confidence ellipse representing the smallest ellipse that covers 95% of the points of the postural sway [mm^2^] (Paillard et al., 2006).

### Non-linear methods

Three non-linear methods were used to measure the regularity of COP signals and compare their sensitivity between the incremented time series and the original time series. Sample Entropy is essentially a negative logarithm of conditional probability of the sequences of a data vector. If a vector of length N has repeated itself in tolerance γ for *m* points, it will also do so for *m*+1 points. The conditional probability means the ratio of counts of repeated time of *m*+1 points to that of *m* points. Thereby, high SE arises from a low probability of repeated sequences in the data. Higher SE means lower regularity and more complexity in the data. On the basis of SE, MSE is a method to evaluate the complexity of signals over different time-scales while MMSE generalizes the analysis to the multivariate case (Wei et al., 2012).

The SE is mathematically computed as follows:

First, from a vector X_*N*_ = {x_1_, x_2_, …, x_N_}, two sequences of *m* consecutive points *X*_*m*_(*i*) = {*x*_*i*_, …, *x*_*i*+*m*−1_} and *X*_*m*_(*j*) = {*x*_*j*_, …, *x*_*j*+*m*−1_} (*i, j* ∈ [1, *N* − *m*], *i* ≠ *j*) are selected to compute the maximum distance and compared to tolerance γ for repeated sequences counting, according to Equation (1). For the sequence *X*_*m*_(*i*), its count is defined as ${\text{B}}_{\text{i}}^{\text{m}}\left(\gamma \right)$.

(1) $$\begin{array}{l} d\left[X_{m}(i),X_{m}(j)\right]\;=\text{ max}[\left|x_{i\;+\;k},x_{j\;+\;k}\right|]\\ \;\leq \gamma (\text{k}\in \left[0,\text{m}-1\right],\gamma \geq 0) \end{array}$$

1 where the tolerance γ equals to 0.1~0.2^*^SD (Richman and Moorman, 2000), SD is the standard deviation of X_N_.

*B*^*m*^(γ) is the average amount of ${\text{B}}_{\text{i}}^{\text{m}}\left(\gamma \right)$ for *i* ∈ [1, *N* − *m*], and *B*^*m*+1^(γ) is the average of *m* +1 consecutive points. Thus, SE is obtained using the Equation (2).

(2) $$\begin{array}{l} SE(N,m,\gamma )\;=\;-ln\left[\frac{B^{m+1}(\gamma )}{B^{m}(\gamma )}\right]\\ \;\;\;\;\;\;\;\;\;\;\;\;\;\;\;\;\;\;\;=-ln\left[\frac{{\left(\text{N}-\text{m}-1\right)}^{-1}{\sum_{\text{i}=1}^{\text{N}-\text{m}-1}\text{B}}_{\text{i}}^{\text{m}+1}\left(\gamma \right)}{{\left(\text{N}-\text{m}\right)}^{-1}{\sum_{\text{i}=1}^{\text{N}-\text{m}}\text{B}}_{\text{i}}^{\text{m}}\left(\gamma \right)}\right] \end{array}$$

MSE has a coarse-grain procedure for the data vector X_N_ before SE computation, which is the main computation difference between MSE and SE. Due to this procedure, MSE is able to measure the distribution of complexity on multiple time-scales, which is fundamentally different from SE. The coarse-grain procedure averages each τ points to generate a new sequence from the data. It is similar to a non-overlapping mean filter with a window length τ. Changing τ leads to sequences in different time-scales. Higher τ means lower frequency components in the sequences. MSE generates the complexity distribution of the data through the sequences in different time-scales, and MSE is equal to SE when τ = 1. The parameter *m* is the length of repeated mode in the data vector, which is defined by the data itself; and the tolerance γ decides the limitation condition of repeated mode. In biological time-series analysis, *m* is typically set at 2 or 3 and γ is 0.15^*^standard deviation (SD) (X_N_) (Costa et al., 2005).

To calculate MSE, the following steps have to be computed: the first step is to form a sequence $y_{j}^{\left(\tau \right)}$ based on the scale factor τ, which can be found using the Equation (3):

(3) $$y_{j}^{\left(\tau \right)}\;=\;\frac{1}{\tau }\sum_{i\;=\;(j\;-\;1)\tau \;+\;1}^{j\tau }x_{i}$$

The equation to compute MSE can be expressed as follows:

(4) $$MSE(N,m,\tau ,\gamma )\;=\;-\ln\left[\frac{A^{m\;+\;1}(\gamma )}{A^{m}(\gamma )}\right]$$

where both *A*^*m*^(γ) and *A*^*m*+1^(γ) are the average repeated amount of two s*equences Y*_*m*_(*i*) and *Y*_*m* + 1_(*i*) [see Equations (1, 2)] to calculate ${\text{B}}_{\text{i}}^{\text{m}}\left(\gamma \right)$) and tolerance γ is also for the formed sequences $y_{j}^{\left(\tau \right)}$ with the length *N*′ = *N*/τ ∈ [10^*m*^, 30^*m*^] (Pincus and Goldberger, 1994). Therefore, τ is defined by length of *N* of *X*_*N*_ and *m*. For example, *N* = 10,000 and *m* = 2,500 as the median of [10^2^, 30^2^] could be selected to calculate the τ_max_ = 10,000/500 = 20 and τ ∈ [1, 20].

MMSE provides a complexity distribution too, not for a data vector but for the matrix vector from multichannel or multivariate data, which is more adaptive to multi-dimension or multi-parameter time series that are routinely measured in experimental and biological systems.

Given the matrix *X*(*p, N*), *p* is the number of channels or variates, *N* is the original length, so that the first step for this matrix is just transforming it into a new matrix *X*′(*p, N*′), based on scale factor τ regardless of *m*, which gives the new matrix in a new time scale like the coarse-grain procedure in MSE. In the second step, *m* and ε are extended to an embedding vector and a time lag vector for a *p*-variate embedded reconstruction respectively. For the consistence of each variate in the matrix, the values of *m* and ε are identical, e.g., *m* = [3,3,3]; ε = [1, 1, 1]. That means three MSE computations with the embedding dimension *m* = 3 for three variates and the time delay ε = 1. The time delay ε = 1 is the best choice when the minimum embedding dimension for each of the time series is 3 (Cao et al., 1998).

In practice its first step is to define temporal scales of the increased length by coarse-graining the *p-variate* ${\left\{x_{k,i}\right\}}_{i=1}^{N},k=1,2,\ldots ,p$. For a scale factor τ, the multivariate coarse-grained time series $y_{k,j}^{\left(\tau \right)}$ is calculated [see Equation (5)], where 1 ≤ *j* ≤ *N*/τ.

(5) $$y_{k,j}^{\left(\tau \right)}\;=\;\frac{1}{\tau }\sum_{i\;=\;(j\;-\;1)\tau +1}^{j\tau }x_{k,i}$$

In order to obtain the MMSE, the multivariate embedded vectors $Y_{m}\left(i\right)\in {\mathbb{R}}^{p}$ must be constructed firstl, which is shown as:

(6) $$\begin{array}{l} Y_{m}(i)\;=\;[y_{1,i},y_{1,i+\varepsilon_{1}},\ldots ,y_{1,i\;+\;(m_{1}-1)\varepsilon_{1}},y_{2,i},y_{2,i+\varepsilon_{2}},\ldots ,\\ \;y_{2,i+\left(m_{2}-1\right)\varepsilon_{2}},\ldots ,y_{p,i},y_{p,i+\varepsilon_{p}},\ldots ,y_{p,i+\left(m_{p}-1\right)\varepsilon_{p}}] \end{array}$$

where $1\leq i\leq {\text{N}}^{\prime }-n\;\text{and}\;{\text{N}}^{\prime }=N/\tau ,\;n=max\left\{M\right\}\times max\left\{\varepsilon \right\}.\;M=\left[m_{1},m_{2},\ldots ,m_{p}\right]\in {\mathbb{R}}^{p}$is the embedding vector, while ε = [ε_1_, ε_1_, …, ε_*p*_] is the time lag vector and $M=\overset{p}{\underset{k=1}{\sum }}m_{k}$. Then the maximum norm is defined by Chebyshev distance between any two composite delay vectors *Y*_*m*_(*i*) and *Y*_*m*_(*j*), that is expressed as:

(7) $$\begin{array}{l} d\left[Y_{m}(i),Y_{m}(j)\right]\;=\;max_{l\;=\;1,\ldots ,m}\\ \;\left\{\left|y\left(i+l-1\right)-y(j+l-1)\right|\right\} \end{array}$$

where *j* ∈ [1, N′ − *n*], *j* ≠ *i*. For a given *Y*_*m*_(*i*), P_i_ is the number of vector pairs that meets *d*[*Y*_*m*_(*i*), *Y*_*m*_(*j*)] ≤ γ, so that $A_{i}^{m}\left(\gamma \right)=P_{i}/\left({\text{N}}^{\prime }-n-1\right)$, where *n* = *max*{*M*} × *max*{τ}. And for all i, $A^{m}\left(\gamma \right)\;=\;{\left({\text{N}}^{\prime }-n\right)}^{-1}\sum_{i=1}^{{\text{N}}^{\prime }-n}A_{i}^{m}\left(\gamma \right)$.

Finally, the average similarity *A*^*m*^(γ) over all *i* ∈ [1, N′ − *n*] and the *A*^*m*+1^(γ) over all *i* ∈ [1, p * (N′ − *n*)] are used to gain the MMSE, as shown in Equation (8).

(8) $$MMSE(N\prime ,M,\tau ,\gamma )\;=\;-ln\left[\frac{A^{m\;+\;1}(\gamma )}{A^{m}(\gamma )}\right]$$

where γ is the tolerance level and N′ is the length of the time series $y_{k,j}^{\left(\tau \right)}$.

The embedding vector $M=\left[m_{1},m_{2},\ldots ,m_{p}\right]\in {\mathbb{R}}^{p}$ and the tolerance level γ in MMSE have the equivalent values with parameters *m* and γ in MSE (Wei et al., 2012).

The three non-linear entropy methods are effective to measure complexity of time series, specifically the SE for the univariate vector, MSE for the univariate vector in multiple time-scale, and MMSE for the multivariate matrix in multiple time-scale respectively. Moreover, it is crucial to have the original time series filtered as all entropy methods are extremely sensitive to random noise. Therefore, our COP data was filtered by a low-pass filter before the computation of entropy.

### Incremental time series

The postural sway was analyzed in the A/P and the M/L direction, COP (A/P) and COP (M/L) respectively. Additional to the original COP data, its increments were calculated 2-fold: First Δ_*inc*_x = [x(t + 1) − x(t − 1)], defined as the ***Increment***; second Δ_*diff*_x = [x(t + 1) − x(t)], defined as ***Difference***. Since mean velocity is a classical parameter of COP estimation, the increments of original COP data, as its equivalent velocity, were created from the original data to remove long-range correlations and avoid potential masking of the complexity of the COP time series.

### Simulation

**Figure 2:**
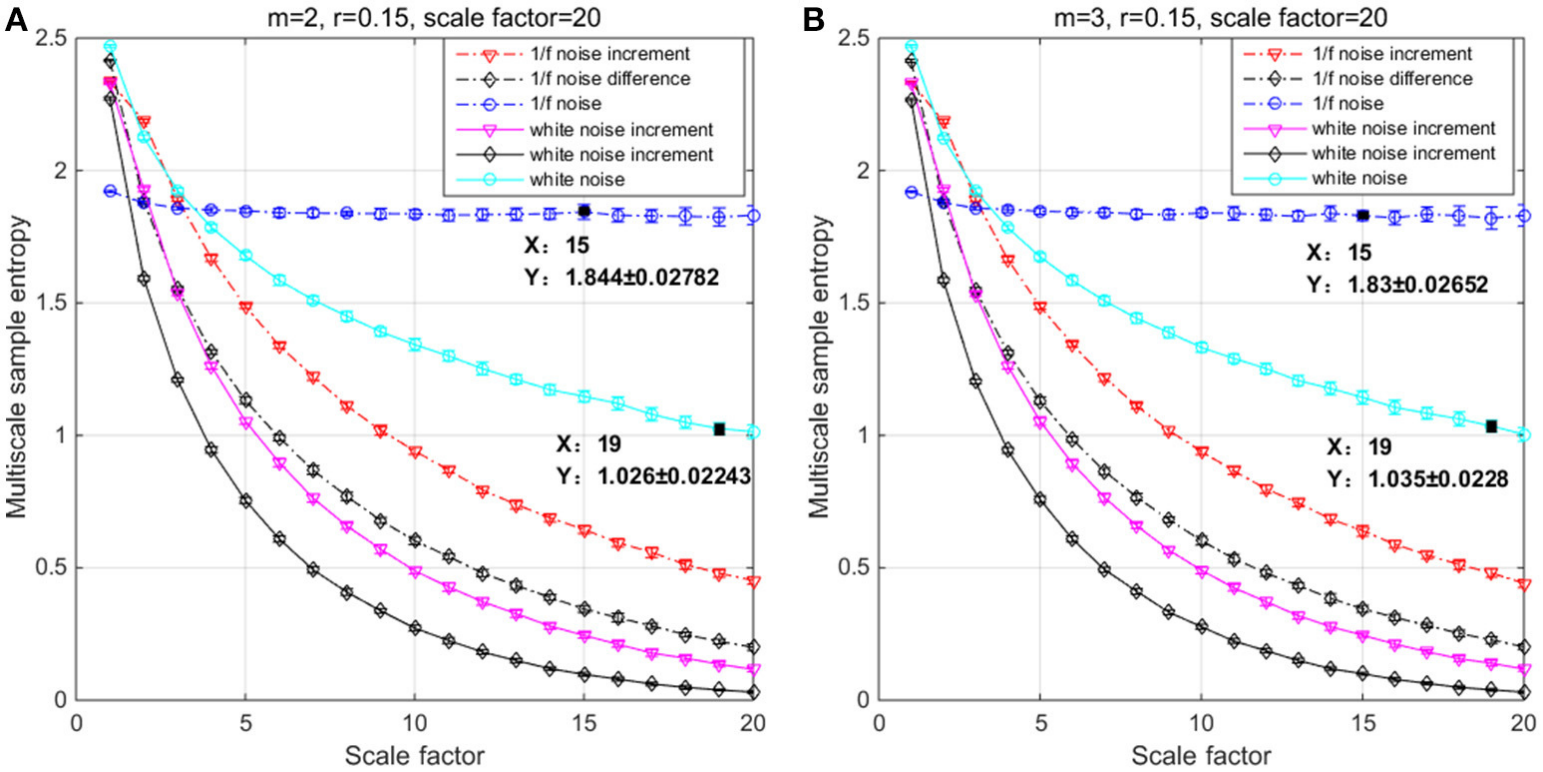
**Multiscale entropy (MSE) analysis of white noise, 1/f noise and their Increment and Difference using (A)** *m* = 2, *r* = 0.15, and τ = 20 and **(B)** *m* = 3, *r* = 0.15, and τ = 20. Each channel has 20 000 data points, and the plots represent an average of 20 independent groups and error bars the standard deviation (SD).

In the simulation, both the original and incremental time series are used to evaluate differences by means of SE, MSE, and MMSE. White noise and 1/f noise are prominent signals to test entropy family measures because of their short-term and long-term correlated properties (Richman and Moorman, 2000; Costa et al., 2005; Ahmed and Mandic, 2011; Wei et al., 2012). To illustrate the behavior of incremental time series and its influence on entropy measures, we considered six time series with a length of *N* = 20,000 (meeting the range minimum $10^{m}\times \tau_{max}$ when *m* = 3 and τ_*max*_ = 20) of each data vector corresponding to 80 s (near to the sum of 30 s in trail and 60 s in rest) of data acquisition at 250 Hz: 1/f noise, 1/f noise ***Increment***, 1/f noise ***Difference***, white noise, white noise ***Increment***, white noise ***Difference***. ***Increment*** and ***Difference*** are computed from both the 1/f noise and the white noise data through Δ_*inc*_x = [x(t + 1) − x(t − 1)] and Δ_*diff*_x = [x(t + 1) − x(t)] respectively. MSE and MMSE were calculated with m = 2 and 3, γ = 0.15^*^SD (Costa et al., 2005) and a scale factor of τ = 1 to 20 (Figure 2) (meeting the range minimum, Richman and Moorman, 2000).

**Figure 3:**
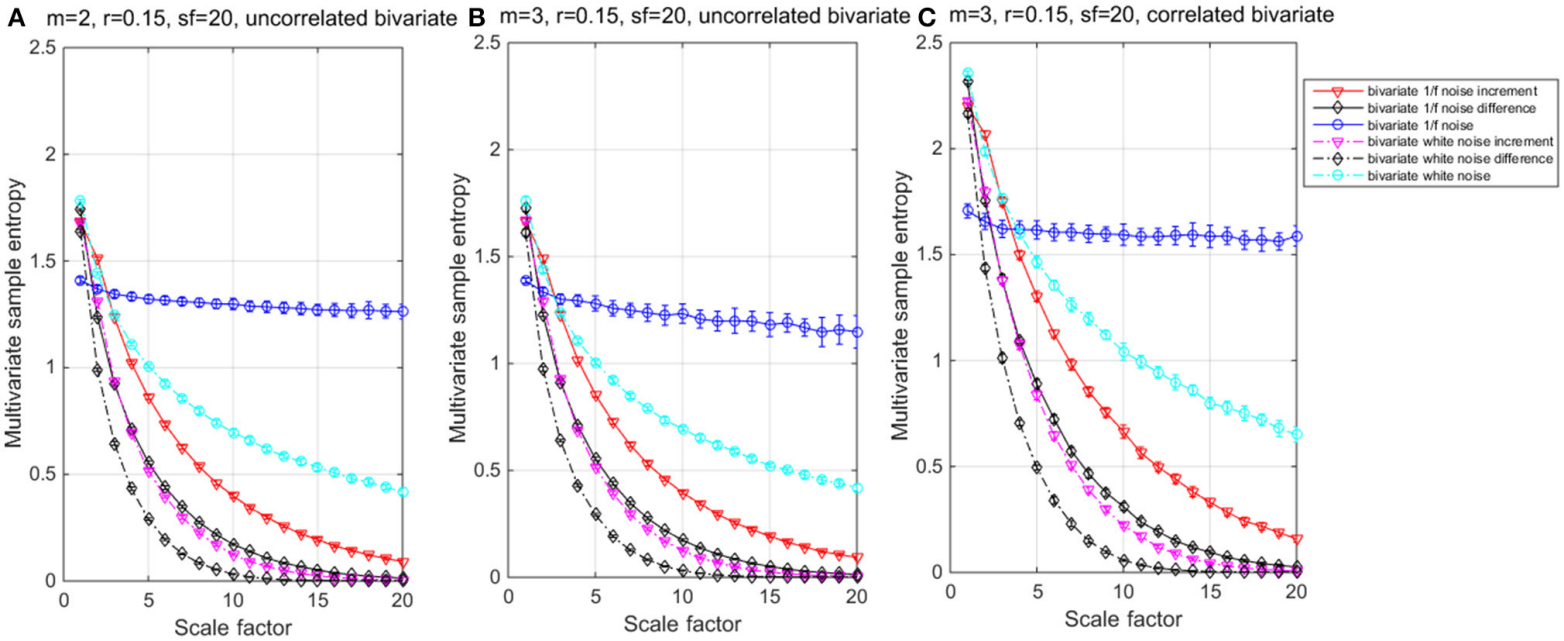
**Multivariate multiscale entropy (MMSE) analysis for uncorrelated bivariate 1/f noise, white noise and their Increment and Difference within ***m*** = 2 (A)** and 3 **(B)** respectively; and for **(C)** correlated bivariate 1/f noise, white noise and their Increment and Difference within *m* = 3.

The results of the simulations show decreasing complexity with increasing scale factor except for the 1/f noise series. To understand the differences between *m* = 2 & 3 on the entropy values, Table 1 shows individual differences as a function of the scale factor for the six considered time series. The simulation results show differences between scale factors 11 and 15 with an absolute deviation of 0.01, indicating that SE is more sensitive on a larger scale with a pattern length of *m* = 3 (Figure 2). The MMSE analysis of bivariate time series shows similar patterns but larger entropy values in the correlated bivariate time series (Figure 3). When computing MSE and MMSE for short-term correlated signals (white noise), a reduction in complexity occurs with larger scale factors in contrast to long-term correlated signals (1/f noise).

**Table 1:** **Differences between the SE calculated with ***m*** = 2 and ***m*** = 3 for different scales and noises**.

**Scale factor**	**1/f noise**			**White noise**		
	**Original**	**Increment**	**Difference**	**Original**	**Increment**	**Difference**
1	0.0010	0.0020	0.0010	0.0000	0.0010	0.0050
2	−0.0020	0.0010	0.0010	0.0010	0.0020	0.0070
3	0.0010	0.0000	−0.0015	0.0010	0.0060	0.0060
4	−0.0020	0.0050	0.0020	0.0010	−0.0010	0.0013
5	0.0020	0.0000	0.0020	0.0040	0.0000	−0.0036
6	−0.0020	−0.0050	0.0057	0.0000	0.0038	0.0010
7	−0.0030	0.0050	0.0045	0.0040	−0.0024	−0.0030
8	0.0020	0.0010	0.0025	0.0080	−0.0010	−0.0025
9	0.0050	0.0010	−0.0027	0.0030	0.0042	0.0056
10	−0.0030	0.0019	0.0000	0.0011	−0.0021	−0.0052
11	−0.0070	0.0028	0.0074	−0.0091	0.0028	0.0017
12	0.0000	−0.0039	−0.0011	−0.0010	0.0000	−0.0046
13	0.0080	−0.0064	0.0000	0.0050	0.0070	−0.0020
14	−0.0010	0.0038	0.0035	−0.0050	0.0018	0.0010
15	0.0140	0.0051	−0.0090	0.0030	−0.0040	−0.0024
16	0.0090	0.0059	−0.0080	0.0015	−0.0070	0.0093
17	−0.0060	0.0010	−0.0019	−0.0030	−0.0050	−0.0022
18	−0.0030	0.0030	−0.0045	−0.0013	0.0022	0.0020
19	0.0030	−0.0070	−0.0059	−0.0090	−0.0035	−0.0038
20	0.0000	0.0011	−0.0017	0.0010	−0.0016	−0.0020

When computing MSE and MMSE for incremented time series with white noise and 1/f noise, reductions of complexity with increasing scales occur. The incremental time series removes the long-term correlated components from the original time series and represent short-term complexity. Compared to the MSE, the MMSE analysis shows lower entropy values per scale and seems to detect signal divergence faster and can, therefore, be considered more suitable for complexity detection.

Based on the simulation results indicating that SE is more sensitive on larger scales, while *m* was set to three, γ = 0.15^*^SD and a scale factor ranging from 1 to 20. A larger scale factor provides a higher resolution of complexity in the time domain and visuo-vestibular regulation is related to low frequencies (0–0.5 Hz) of the COP displacement (Dichgans et al., 1976; Paillard et al., 2006).

### Statistical analysis

Friedman tests were used to examine the effect of condition (i.e., EO, EC, PV) on all postural parameters. The complexity index (CI), defined as the integral of the MSE or MMSE curve, was used for the statistical analysis of the entropy measures (MSE, MMSE). The normality of the data sets was verified using the Kolmogorov-Smirnov test. *Post-hoc* pairwise comparisons (between-conditions) were performed when needed using Wilcoxon's tests with a Bonferroni adjustment. The effect size values were described by the magnitude of change expressed as Cliff's delta (|*r*|). All tests were performed using IBM SPSS Statistics version 16 with a level of significance set at *p* < 0.05.

## Results

The Kolmogorov-Smirnov tests indicated non-parametric distribution of the data sets and therefore the data is reported using median (*Med*) and inter-quartile range (*IQR*). For the classical parameters, Friedman tests indicated significant differences in path length, mean velocity and mean frequency (A/P), depending on the condition (Table 2). The *post-hoc* Wilcoxon's analyses revealed a statistically significant difference between the EO and EC conditions for path length, [EC (*Med* = 230.95, *IQR* = 120.52) and EO (*Med* = 184.19, *IQR* = 53.62), *z* = 2.934, *p* ≤ 0.01], mean velocity [EC (*Med* = 7.7, *IQR* = 4.02) and EO (*Med* = 6.14, *IQR* = 1.78), *z* = 2.934, *p* ≤ 0.01], and mean frequency [EC (*Med* = 0.52, *IQR* = 0.31) and EO (*Med* = 0.45, *IQR* = 0.30), *z* = 2.934, *p* ≤ 0.01]. The small effect size calculation for the tree parameters with Cliff's delta values (*r* < 0.35) suggests minimal practical significance. For the 95% confidence ellipse, mean frequency (M/L), root-mean-square in A/P, or M/L direction (Table 2) no differences were found.

**Table 2:** **Results of non-parametric statistical tests (median ± IQR) on differences between the three experimental conditions (EC, PV, EO) on classical and non-linear complexity parameters**.

					**Friedman tests**			**Wilcoxon's** ***post-hoc*** **comparisons**				
	**Classical parameters**						**EC vs. EO**		**EC vs. PV**		**PV vs. EO**	
	**EC**	**PV**	**EO**	**Trend**	***χ^2^*(2)**	***p***	***z***	**|r|**	***z***	**|r|**	***z***	**|r|**
Path Length	230.95 ± 120.52	208.14 ± 76.33	184.19 ± 53.62	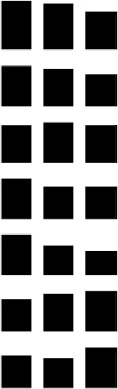	14.73	**0.001**	2.934	0.273^**^				
Mean Velocity	7.7 ± 4.02	6.94 ± 2.54	6.14 ± 1.78		14.73	**0.001**	2.934	0.273^**^				
Confidence Ellipse	75.77 ± 61.27	84.32 ± 120.52	77.81 ± 43.34		0.55	0.761						
Mean Frequency (A/P)	0.52 ± 0.31	0.42 ± 0.33	0.45 ± 0.3		10.36	**0.006**	2.934	0.339^**^				
Mean Frequency (M/L)	0.26 ± 0.13	0.2 ± 0.08	0.15 ± 0.11		5.09	0.078						
RMS (A/P)	1.11 ± 0.66	1.19 ± 0.85	1.3 ± 0.5		0.55	0.761						
RMS (M/L)	3.79 ± 2.22	3.34 ± 2.38	4.4 ± 2.44		0.55	0.761						
	**Original time series**				*χ^2^***(2)**	***p***	***z***	**|r|**	***z***	**|r|**	***z***	**|r|**
SE (A/P)	1.28 ± 0.16	1.30 ± 0.15	1.32 ± 0.13	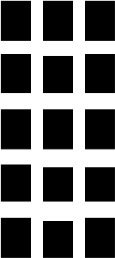	0.55	0.761						
SE (M/L)	0.76 ± 0.28	0.72 ± 0.16	0.77 ± 0.46		2.91	0.234						
CI_MSE (A/P)	18.99 ± 2.4	19.4 ± 2.26	19.14 ± 1.47		6.73	**0.035**						
CI_MSE (M/L)	12.49 ± 4.26	11.46 ± 2.09	11.07 ± 6.13		1.27	0.529						
CI_MMSE	13.98 ± 1.15	13.58 ± 1.12	14.17 ± 0.56		6.73	**0.035**					2.401	0.818^*^
	**Incremented time series**				*χ^2^***(2)**	***p***	***z***	**|r|**	***z***	**|r|**	***z***	**|r|**
SE (A/P)	2.35 ± 0.02	2.34 ± 0.01	2.34 ± 0.01	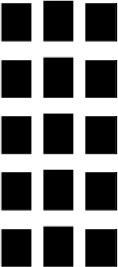	5.09	0.078						
SE (M/L)	2.36 ± 0.01	2.35 ± 0.01	2.35 ± 0.02		4.55	0.103						
CI_MSE (A/P)	18.91 ± 1.14	18.29 ± 1.35	17.99 ± 1.73		18.727	**0.000**	2.934	−0.025^**^	2.401	1.000^*^	2.934	0.669^**^
CI_MSE (M/L)	20.29 ± 1.37	19.94 ± 0.84	19 ± 0.95		8.419	**0.015**	2.490	0.124^*^	2.701	0.950^**^		
CI_MMSE	9.03 ± 0.4	8.83 ± 0.43	8.46 ± 0.71		14.727	**0.001**	2.934	0.008^**^	2.667	0.967^**^	2.578	0.752^*^
	**Difference time series**				*χ^2^***(2)**	***p***	***z***	**|r|**	***z***	**|r|**	***z***	**|r|**
SE (A/P)	2.27 ± 0.01	2.27 ± 0.01	2.27 ± 0.01	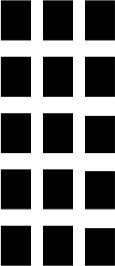	7.47	**0.024**						
SE (M/L)	2.27 ± 0.01	2.27 ± 0.01	2.26 ± 0.01		3.82	0.148						
CI_MSE (A/P)	12.29 ± 0.84	11.94 ± 0.85	11.62 ± 1.14		17.636	**0.001**	2.934	−0.074^**^			2.934	0.686^**^
CI_MSE (M/L)	13.2 ± 0.99	12.88 ± 0.69	12.03 ± 0.65		8.727	**0.013**	2.534	−0.074^*^	2.667	0.950^**^		
CI_MMSE	5.24 ± 0.23	5.15 ± 0.19	5 ± 0.23		16.233	**0.000**	2.934	−0.091^**^	2.497	1.000^*^	2.801	0.669^**^

*EC, eyes closed condition; PV, perturbed vision; EO, eyes open condition. A/P, anterior-posterior; M/L, medio-lateral. SE, Sample Entropy; CI_MSE, complexity index of Multi-Scale Entropy; CI_MMSE, complexity index of Multivariate Multi-Scale Entropy*.

* *p < 0.05 (after Bonferroni correction, p < 0.017)*,

** *p < 0.01. Bold values shows significantly different among three conditions*.

The analysis for ***Original*** time series showed a significant effect of condition on CI_MSE (A/P) and CI_MMSE (Table 2). The *post-hoc* Wilcoxon's analyses revealed a statistically significant difference between the EO and PV conditions for CI_MMSE, [EO (*Med* = 13.98, *IQR* = 1.15) and PV (*Med* = 13.58, *IQR* = 1.12), *z* = 2.401, *p* ≤ 0.05]. The large effect size calculation with a Cliff's delta value (*r* > 0.80) suggests high practical significance.

For the ***Incremented*** time series, significant differences between conditions were found in three of the five parameters (Table 2). The *post-hoc* Wilcoxon's analyses revealed a statistically significant difference between the EC and EO conditions for CI_MSE (A/P) [EC (*Med* = 18.91, *IQR* = 1.14) and EO (*Med* = 17.99, *IQR* = 1.73), *z* = 2.934, *p* ≤ 0.01], CI_MSE (M/L) [EC (*Med* = 20.29, *IQR* = 1.37) and EO (*Med* = 19.00, *IQR* = 0.95), *z* = 2.490, *p* ≤ 0.05], and CI_MMSE [EC (*Med* = 9.03, *IQR* = 0.40) and EO (*Med* = 8.46, *IQR* = 0.71), *z* = 2.934, *p* ≤ 0.01]. The small effect size calculation for the tree parameters with Cliff's delta values (*r* < 0.35) suggests minimal practical significance.

When comparing the EC and PV conditions, the *post-hoc* Wilcoxon's analyses revealed a statistically significant differences for CI_MSE (A/P) [EC (*Med* = 18.91, *IQR* = 1.14) and PV (*Med* = 18.29, *IQR* = 1.35), *z* = 2.401, *p* ≤ 0.05], CI_MSE (M/L) [EC (*Med* = 20.29, *IQR* = 1.37) and PV (*Med* = 19.94, *IQR* = 0.84), *z* = 2.701, *p* ≤ 0.01], and CI_MMSE [EC (*Med* = 9.03, *IQR* = 0.40) and PV (*Med* = 8.83, *IQR* = 0.43), *z* = 2.667, *p* ≤ 0.01]. The large effect size calculation for the three parameters with Cliff's delta values (*r* > 0.95) suggests large practical significance.

When comparing the PV and EO conditions, the *post-hoc* Wilcoxon's analyses revealed statistically significant differences for CI_MSE(A/P) [PV (*Med* = 18.29, *IQR* = 1.35) and EO (*Med* = 17.99, *IQR* = 1.73), *z* = 2.934, *p* ≤ 0.01], and CI_MMSE [PV (*Med* = 7.7, *IQR* = 4.02) and EO (*Med* = 6.14, *IQR* = 1.78), *z* = 2.578, *p* ≤ 0.05]. The medium effect size calculation for the two parameters with Cliff's delta values (*r* > 0.65) suggests high practical significance.

In the ***Difference*** time series, four of the five non-linear parameters significantly changed between conditions (Table 1). The *post-hoc* Wilcoxon's analyses revealed statistically significant differences between the EO and EC conditions for CI_MSE (A/P) [EC (*Med* = 12, 29 *IQR* = 0.84) and EO (*Med* = 11.62, *IQR* = 1.14), *z* = 2.934, *p* ≤ 0.01], CI_MSE (M/L) [EC (*Med* = 13.20, *IQR* = 0.99) and EO (*Med* = 12.03, *IQR* = 0.65), *z* = 2.534, *p* ≤ 0.05], and CI_MMSE [EC (*Med* = 5.24, *IQR* = 0.23) and EO (*Med* = 5.00, *IQR* = 0.23), *z* = 2.934, *p* ≤ 0.01]. The small effect size calculation for the tree parameters with Cliff's delta values (*r* < 0.35) suggests minimal practical significance.

When comparing the EC and PV conditions, the *post-hoc* Wilcoxon's analyses revealed a statistically significant differences for CI_MSE (M/L), [EC (*Med* = 13.20, *IQR* = 0.99) and PV (*Med* = 12.88 *IQR* = 0.69), *z* = 2.667, *p* ≤ 0.01], and CI_MMSE [EC (*Med* = 5.24, *IQR* = 0.23) and PV (*Med* = 5.15, *IQR* = 0.19), *z* = 2.497, *p* ≤ 0.05]. The large effect size calculation for the three parameters with Cliff's delta values (*r* > 0.90) suggests large practical significance.

When comparing the PV and EO conditions, the *post-hoc* Wilcoxon's analyses revealed statistically significant differences for CI_MSE (A/P), [PV (*Med* = 11.94, *IQR* = 0.85) and EO (*Med* = 11.62, *IQR* = 1.14), *z* = 2.934, *p* ≤ 0.01], and CI_MMSE [PV (*Med* = 5.15, *IQR* = 0.19) and EO (*Med* = 5.00, *IQR* = 0.23), *z* = 2.801, *p* ≤ 0.01]. The medium to large effect size calculation for the two parameters with Cliff's delta values (*r* > 0.60) suggests large practical significance.

## Discussion

In this experiment, classical parameters were tested against non-linear complexity parameters to reveal differences between the COP displacements during three standing conditions. Non-linear entropy family methods resulted in better uncovering of postural displacement differences when compared to the classical posturographic methods. Only three of the seven classical parameters [i.e., PL, MV, MF (A/P)] were able to differentiate between conditions (EO, EC, PV). Amongst the five studied entropy measures, only two showed significant differences between conditions [i.e., CI_MSE (A/P) and CI_MMSE] using the ***Original*** time series, and in the ***Incremented*** and ***Difference*** time series CI_MSE (A/P), CI_MSE (M/L) and CI_MMSE showed higher sensitivity to the condition effect. Between-conditions differences in the ***Original*** time series were only revealed for the CI_MMSE parameter. In the ***Increment*** and ***Difference*** time series between-conditions differences were found for CI_MSE (A/P), CI_MSE (M/L) and CI_MMSE parameters. The most consistent parameter for the three time series is the CI_MMSE and could therefore be considered as the most reliable parameter in detecting between-conditions differences.

For visual feedback, the results show that the complexity quantified by MSE, and MMSE statistic in ***Increment*** and ***Difference*** is lower in the EC condition compared to EO and PV. Even though the statistical analysis has not shown significant differences on the COP displacement related to the effect of visual feedback, the entropy values for SE (A/P) of the ***Original*** is lowest in the EC condition. Higher entropy values are related to more irregularity, something that may be associated with a functional decline of the postural control system resulting in maladaptive responses to perturbations and thereby destabilizing the balance control (Vaillancourt and Newell, 2002; Schniepp et al., 2013). This finding is consistent with previous research (Roerdink et al., 2011) showing that physical and physiological visual parameters affect postural control during quiet standing and therefore the COP displacement (cf. Stins et al., 2009; Vuillerme and Pinsault, 2009). The sample size and the choice of young healthy participants that were recruited from the Sports Science department may have influenced the results as they may have higher postural capacities compared to age matched subjects. Working with healthy young adults increases the difficulty to distinguish between EO and EC compared to older adults (Prieto et al., 1996). Taking this into account the relation between COP regularity and the amount of attention invested in posture (Donker et al., 2007; Stins et al., 2009), are in line with the findings of our study. The COP regularity in the ***Original*** time series showed no differences between conditions by means of sample entropy while in the ***Difference*** time series such differences were uncovered. This confirms previous work showing that a decrease of the complexity of the physiological and behavioral systems is observed when the kinesthetic cues are reduced (Newell et al., 1997) but also that incremented time series provide short-term correlated components containing more information about the non-linear system (Ramdani et al., 2009).

Using entropy measures allows us to deal with highly irregular and variable signals like postural sway (Prieto et al., 1996). However, limitations of the particular parameters and properties of the entropy measures have to be discussed. MSE is an entropy measure for univariate time series and MMSE is extended to multivariate cases. Even though the results of MSE and multivariate MSE analysis are promising, some problems still have to be resolved when working with postural data. Entropy measures assess the complexity of physiological time series signals rather than measure motor performance. The creation of one complexity index summing all twenty scales needs to be addressed in future research as it is currently unknown which time-scale is more important or related to postural control. Data length affects entropy measures but it depends on the experiment and therefore a high sampling rate of 250 Hz was chosen to obtain the necessary data length. The COP signal does not contain high frequency signals and the use of a low-pass filter (cut-off 5 Hz) is common. This may create an oversampling issue and the first several scales may not have physiological meaningful information related to dynamical COP changes. Furthermore, the coarse-graining procedure reduced the input data length to half its original size for each successive data scale and therefore potentially changed the intrinsic dynamical scales defined by the signal-generating system.

The reduction of MSE with increasing time-scale is based on the tolerance settings (i.e., constant fraction of the variance of the original time series) and the conventional γ^*^SD tolerance could be replaced by the variance of each coarse grained time series (Humeau-Heurtier, 2015).

Previous research from our laboratory has shown those MSE characteristics and a possible solution could be the use of diverse empirical mode decomposition techniques (Shih et al., 2015). Decomposing the ***Original*** time series into intrinsic mode functions when testing different combinations of frequency bands with relevant complexity indexes has been shown to be efficient and promising in studies on elderly subjects exposed to slight vibrations under their feet (Wei et al., 2012). Empirical mode decomposition and Hilbert–Huang transformation can also be used to observe the physiological signals in instantaneous frequency and instantaneous amplitude through intrinsic mode functions, and then transformed inversely into the time domain to observe the changes in different conditions (Shih et al., 2015). However, the ***Increment*** and ***Difference*** time series approaches also seem appropriate as they removed trends from the original time series and showed changes in the COP displacements. Other approaches (Yeh et al., 2016) such as the above-mentioned empirical mode decomposition also provide useful data-driven scale factor and intrinsic entropy calculation of complex time series showing the possibility to explore the dynamical complexity of postural control in the future by combining incremented time series and empirical mode decomposition methods.

## Conclusions

The current work supports the notion that some measures of non-linear entropy discriminate postural displacement better than classical measures when using incremented time series. Future studies should look to extend applied methods by examining other populations with impaired motor control. We also suggest that future research should consider empirical mode decomposition in combination with incremented time series across different clinical conditions to establish the reliability and validity of this new approach.

## Author contributions

CH, LM, PF, and BI designed and conducted the experiment, QW and JS conducted the analysis and simulations, CH, QW, and JS wrote the manuscript.

### Conflict of interest statement

The authors declare that the research was conducted in the absence of any commercial or financial relationships that could be construed as a potential conflict of interest.
